# A Homozygous Missense *COL1A1* Variant (p.Glu684Lys) Associated with an Arthrochalasia-like Ehlers–Danlos Syndrome Phenotype: A Case Report

**DOI:** 10.3390/genes17060679

**Published:** 2026-06-10

**Authors:** Tatiana Markova, Evgeniya Melnik, Maksim Kurelev, Tatiana Cherevatova, Alexandra Nikolaeva, Daria Gorodilova, Nina Demina, Elena Dadali

**Affiliations:** Research Centre for Medical Genetics, 115522 Moscow, Russia

**Keywords:** arthrochalasia Ehlers–Danlos syndrome, aEDS, arthrochalasia-like EDS phenotype, *COL1A1*, autosomal recessive, muscle hypotonia, congenital hip dislocation, COL1-related overlap disorder, C1ROD

## Abstract

Background/Objectives: Arthrochalasia Ehlers–Danlos syndrome (aEDS) is a rare connective tissue disorder characterized by severe joint hypermobility, congenital hip dislocation, skin hyperextensibility, and muscle hypotonia. It is typically caused by heterozygous splice-site variants in *COL1A1* or *COL1A2*, leading to exon 6 skipping. Autosomal recessive forms are extremely rare and have been reported predominantly in families from Saudi Arabia carrying the homozygous *COL1A1* missense variant c.2050G>A, p.(Glu684Lys), with clinical presentations ranging from severe to mild. Methods: Clinical and molecular genetic evaluation of the patient was performed. Whole-exome sequencing was carried out, followed by confirmatory Sanger sequencing in the proband and both parents. Results: A 10-month-old boy presented with severe congenital hypotonia, bilateral hip dislocation, generalized joint hypermobility, skin hyperextensibility and craniofacial dysmorphism. A homozygous likely pathogenic variant NM_000088.4:c.2050G>A, p.(Glu684Lys) was identified in exon 31 of *COL1A1*; both healthy parents were confirmed to be heterozygous carriers of this variant. To our knowledge this is the first reported case in the Russian population and one of the few cases described worldwide of an autosomal recessive arthrochalasia-like EDS phenotype. Conclusions: This case further refines the phenotypic characterization associated with the recurrent homozygous *COL1A1* p.(Glu684Lys) variant, demonstrating an arthrochalasia-like EDS phenotype of intermediate severity between the severe neonatal form with respiratory distress and recurrent fractures and the classical EDS. It further highlights the importance of considering collagenopathies in the differential diagnosis of congenital hypotonia, particularly in cases initially suggestive of neuromuscular disorders.

## 1. Introduction

Ehlers–Danlos syndromes (EDS) comprise a group of genetically heterogeneous inherited connective tissue disorders characterized by marked clinical variability. The core features include generalized joint hypermobility and skin hyperextensibility, as codified in the revised Villefranche nosology by Beighton et al. in 1998 [1]. According to the current international classification, 13 EDS subtypes are recognized, caused by pathogenic variants in 20 genes encoding structural components of connective tissue and enzymes involved in their biosynthesis and processing [2,3].

Despite the availability of an established classification, pathogenic variants in known genes are not identified in a subset of patients with a clinical phenotype consistent with EDS, suggesting broader genetic heterogeneity [4]. In addition, individuals with confirmed molecular diagnoses show considerable clinical variability, often including features overlapping neuromuscular disorders. These observations highlight the need for further characterization of the clinical and genetic features of individual EDS subtypes, particularly rare ones, which remain diagnostically challenging. One such subtype is arthrochalasia EDS (aEDS), caused by pathogenic variants in the *COL1A1* and *COL1A2* genes encoding the pro-α1 and pro-α2 chains of type I collagen, respectively.

Notably, variants in *COL1A1* and *COL1A2* are associated not only with aEDS but also with a spectrum of related conditions, including osteogenesis imperfecta (OI), Caffey disease, the classical and cardiac-valvular forms of EDS, presenting with overlapping clinical features. Given this phenotypic continuum, Morlino et al. (2020) proposed the term “COL1-related overlap disorder” (C1ROD) to define a distinct entity representing a bridging phenotype between EDS and OI, typically characterized by predominant EDS features with or without mild OI manifestations [5].

The molecular basis of aEDS caused by variants in *COL1A1* and *COL1A2* is relatively homogeneous and involves pathogenic variants leading to complete or partial skipping of exon 6, resulting in defective N-propeptide processing of type I procollagen [6]. Most cases follow an autosomal dominant inheritance pattern. However, several recent reports, primarily from Saudi Arabia, have described families with autosomal recessive inheritance due to a recurrent homozygous *COL1A1* missense variant c.2050G>A, p.(Glu684Lys), showing heterogeneous clinical presentation [7,8,9]. This variability, together with the rarity of the autosomal recessive form of *COL1A1*-related EDS, complicates diagnosis. Therefore, each additional case contributes to refining the clinical spectrum and improving differential diagnostic criteria for related conditions with overlapping features.

The aim of this study is to report the clinical features of the Russian patient with autosomal recessive arthrochalasia-like Ehlers–Danlos syndrome phenotype caused by the homozygous *COL1A1* variant (NM_000088.4):c.2050G>A, p.(Glu684Lys), representing one of the few cases described worldwide, and to further illustrate the phenotypic variability associated with this variant.

## 2. Materials and Methods

### 2.1. Clinical Evaluation

A comprehensive examination was conducted on a 10-month-old male proband with phenotypic signs of connective tissue disorder in combination with severe muscle hypotonia and facial dysmorphisms. To clarify the diagnosis, clinical-genealogical analysis, biochemical blood analysis, echocardiography, radiography of the hip joints and long bones of the limbs, and molecular genetic testing were used.

### 2.2. Molecular Genetic Testing

Whole-exome sequencing (WES) was performed. Genomic DNA was extracted from blood samples of the proband and his healthy parents using standard methods with the QIAamp DNA Blood Mini Kit (Qiagen, Hilden, Germany).

DNA analysis of the proband was performed on a DNBSEQ-400 next-generation sequencer (MGI Tech Co., Ltd., Shenzhen, China) using paired-end reading (2 × 150 bp). Sample preparation utilized the methodology of selective capture of DNA regions corresponding to the coding areas of approximately 20,000 genes, using technology for creating circular NGS libraries (VAHTS Universal Plus DNA Library Prep kit for MGI V2 NDM627-01 and VAHTS Target Capture Hybridization and Wash Kit, Vazyme, Nanjing, China).

Sequencing data analysis was performed using the NGS-data-Genome program developed in the Information and Analytical Department of the FSBSI “RCMG” (registration number 2021662119). The average coverage depth was ~95×, with less than 1.92% of target fragments having coverage below <10×.

For pathogenicity assessment, the allele frequency of variants was annotated using population databases (1000 Genomes Project, gnomAD v3.1.2), and clinical significance was determined by reviewing the Online Mendelian Inheritance in Man (OMIM) database and relevant scientific literature.

Pathogenicity classification was performed in strict accordance with the recommendations of the American College of Medical Genetics and Genomics (ACMG).

To assess familial segregation, Sanger sequencing was conducted for the proband and parents. Sequencing was carried out on an ABI Prism 3500 Genetic Analyzer (Applied Biosystems, Foster City, CA, USA) according to the manufacturer’s protocol. The following primers for the PCR were selected: Forward: 5′-CACCCCACACCCTATCTCCAT-3′, Reverse: 5′-CTCTCTCGCTGCATCCGTC-3′. The size of the amplified segment for Sanger sequencing is 295 base pairs. The identified variant is described according to the MANE Select transcript NM_000088.4, following standard HGVS nomenclature guidelines [10].

## 3. Results

The proband is a 10-month-old boy, the first child of healthy consanguineous parents of Avar ethnicity. The pregnancy was complicated by oligohydramnios and breech presentation. He was delivered by cesarean section at 39 weeks of gestation with a birth weight of 3100 g (−0.62 SDS), length of 50 cm (0.2 SDS), and Apgar scores of 6 at 1 min and 7 at 5 min. At birth, dolichocephaly, microretrognathia, severe generalized muscle hypotonia, hyporeflexia, generalized joint hypermobility (more pronounced in the distal extremities), bilateral congenital hip dislocation, and skin hyperextensibility were observed. Echocardiography revealed mitral valve prolapse with hemodynamically insignificant mitral regurgitation (grade 1+). Quantitative echocardiographic parameters, including valve leaflet thickness, regurgitant fraction or jet area, and left atrial dimensions, were not available from the clinical record. Creatine phosphokinase levels were within the normal range (131 U/L). At 3 months of age, the patient developed bilateral pneumonia without requiring respiratory support. Motor development was markedly delayed: by 10 months, he was able to hold his head inconsistently and roll onto his side. The first syllables appeared at 9 months. In the context of severe hypotonia and marked motor developmental delay, this was regarded as part of an overall neurodevelopmental delay. However, formal cognitive assessment at 10 months was limited by the severity of motor impairment.

**Figure 1:**
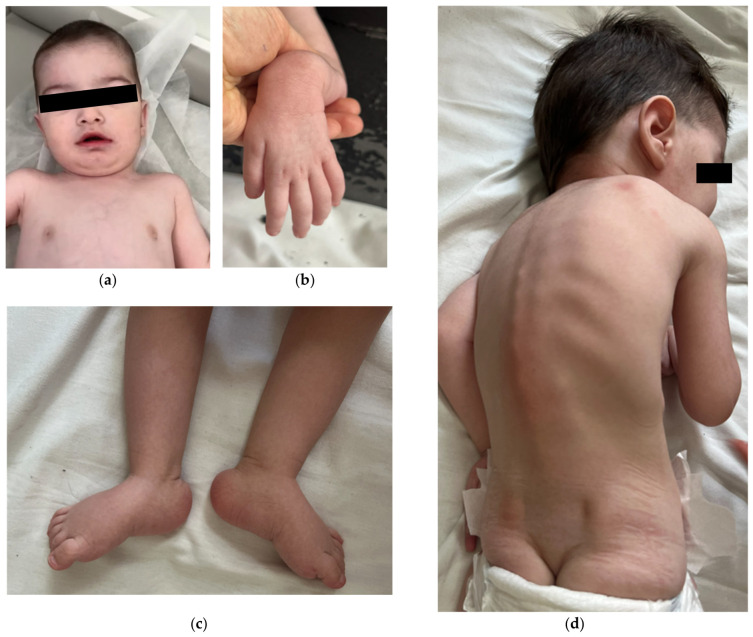
Clinical features: (**a**) Facial phenotype: dolichocephaly, high forehead, telecanthus, broad nasal bridge, eyelid puffiness, retrognathia, low-set ears, blue sclerae, prominent vascular pattern on the trunk. (**b**) Joint laxity at the wrist. (**c**) Protruding fleshy heels; joint laxity at the ankles. (**d**) Thoracolumbar kyphoscoliosis.

At 10 months of age, the patient’s length was 78 cm (+1.34 SD), weight 7.5 kg (−2.45 SD), and head circumference 45.5 cm (−0.33 SD). Craniofacial features included dolichocephaly, a high forehead, telecanthus, a broad nasal bridge, periorbital puffiness, retrognathia, low-set ears, and blue sclerae. Additional findings comprised prominent superficial vasculature on the trunk, soft and hyperextensible skin, wrinkled skin, fleshy heel pads, diffuse muscle hypotonia, hyporeflexia, and generalized muscle weakness, more pronounced distally (2/5 on the Medical Research Council scale vs. 3/5 proximally). Generalized joint hypermobility was evident, with “floppy” wrists and feet (Beighton score 8/9), as well as thoracolumbar kyphoscoliosis. The patient demonstrated poor head control and was able to sit with support only briefly. No bulbar or respiratory involvement was observed (Figure 1).

Given the presence of congenital hip dislocation, severe joint hypermobility, dysmorphic features, generalized muscle hypotonia, and hyporeflexia in the male proband, the differential diagnosis was focused on congenital muscular dystrophies and Ehlers–Danlos syndromes.

**Figure 2:**
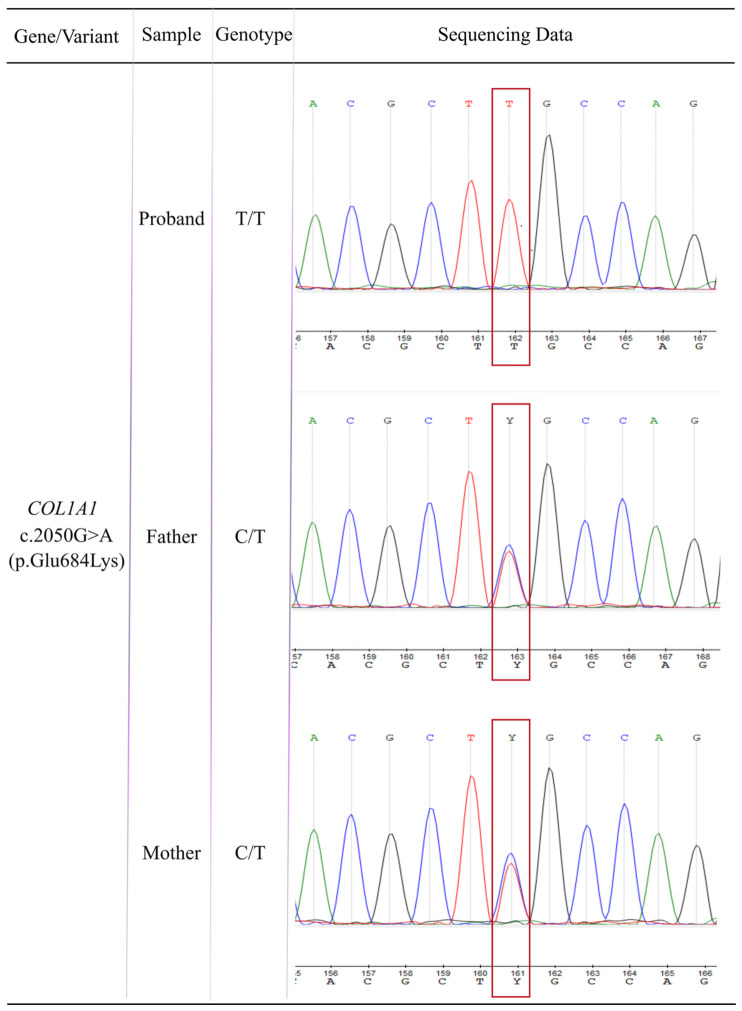
Results of Sanger sequencing (reverse/antisense strand). The proband shows T/T, the parents show C/T. The variant is described as c.2050G>A in the *COL1A1* gene on the sense strand; the complementary bases are shown due to reverse strand analysis. The variant is indicated by a red frame.

Whole-exome sequencing revealed a previously described nucleotide sequence variant in exon 31 of the *COL1A1* gene (NM_000088.4) c.2050G>A (p.(Glu684Lys)) in a homozygous state. The identified variant was confirmed in both directions in the proband by Sanger sequencing and was inherited from both healthy parents (Figure 2).

The chromatograms above display a sequence of the *COL1A1* gene in coding exon 31, revealing a biallelic variant, c.2050G>A (p.Glu684Lys), in the proband.

The variant NM_000088.4:c.2050G>A, p.(Glu684Lys) was classified as Likely Pathogenic in the present study according to the ACMG/AMP 2015 [11] guidelines based on the following criteria: PM1 (moderate)—located in a well-established functional domain without benign variation, with pathogenic missense variants reported in ClinVar (Variation IDs: 808290, 4081255, 4282345); PM2 (moderate)—very low population frequency (0.0007% in gnomAD v4.1.1); PP3 (supporting)—multiple bioinformatic tools predict a damaging effect, although in silico predictions are not independent and require experimental validation; and PP5 (supporting)—previously reported as pathogenic by a reputable source, though the evidence underlying that classification was not provided and may not be easily obtainable. Additionally, the variant has been independently identified in multiple unrelated families of Saudi Arabian origin, further supporting its pathogenic role [7,8]. The combination of two moderate (PM1 and PM2) and two supporting (PP3 and PP5) criteria meets the ACMG/AMP 2015 recommendation for a Likely Pathogenic classification [11].

Based on the molecular genetic findings and the clinical presentation, a diagnosis consistent with an autosomal recessive arthrochalasia-like Ehlers–Danlos syndrome phenotype was established.

## 4. Discussion

Arthrochalasia Ehlers–Danlos syndrome type 1 (aEDS type 1) is a rare disorder caused by pathogenic variants in the *COL1A1* gene. The OMIM database currently recognizes only the autosomal dominant form of aEDS (OMIM:130060). However, several reports describe families with an autosomal recessive pattern of inheritance with the recurrent homozygous *COL1A1* variant c.2050G>A, p.(Glu684Lys). The first such cases were reported by Alazami et al. (2016) in two unrelated patients from Saudi Arabia, who presented with severe neonatal manifestations including respiratory distress, profound hypotonia, congenital joint dislocations, and recurrent fractures [7]. Craniofacial dysmorphism was also noted [7]. In contrast, Almatrafi et al. (2020) described a family with the same variant but a milder phenotype resembling classical EDS, characterized predominantly by skin hyperextensibility and fragility [8]. More recently, Marchant et al. (2024) identified a family with this variant in a cohort of patients with suspected neuromuscular disorders, although only limited clinical data were provided [9]. Taken together, these findings indicate substantial phenotypic variability in autosomal recessive *COL1A1*-related EDS (Table 1).

To our knowledge, the present case represents the first reported instance of arthrochalasia-like EDS phenotype in the Russian population. Similar to previously described patients, our proband presented with severe congenital hypotonia and delayed psychomotor development. However, in contrast to the more severe neonatal forms described by Alazami et al. (2016) [7], where respiratory distress required immediate intervention from birth, our patient had Apgar scores of 6 and 7 and did not require respiratory support; bilateral pneumonia at 3 months resolved without progression to chronic respiratory involvement, and no fractures were observed throughout the follow-up period. Motor milestones remained markedly delayed at 10 months, with inconsistent head control and brief supported sitting, consistent with the hypotonia severity seen in the Alazami cohort but without the associated respiratory dependence. At the same time, the clinical presentation was more severe than the classical EDS-like picture reported by Almatrafi et al. (2020), in whom neonatal involvement was entirely absent and cutaneous features predominated [8]. The phenotype observed in our patient can be considered to fall within the arthrochalasia-like EDS spectrum, representing a form of intermediate clinical severity between the severe neonatal presentation and milder classical EDS-like manifestations. This phenotypic variability highlights the complexity of genotype-phenotype correlations in type I collagenopathies and supports the concept of a phenotypic continuum. It must be noted, however, that longitudinal follow-up data remain unavailable across all reported cases, including ours, and further observation is required to determine whether this intermediate phenotype stabilizes or carries risk of progressive deterioration over time. In this context, the term “COL1-related overlap disorder” proposed by Morlino et al. (2020) may be applicable to such cases [5].

From a clinical perspective, this case emphasizes the importance of including connective tissue disorders in the differential diagnosis of infants presenting with congenital hypotonia and hyporeflexia. Such presentations may mimic primary neuromuscular disorders and lead to diagnostic delays [12]. Careful clinical evaluation, including assessment of joint hypermobility, congenital dislocations, and skin involvement, may help guide appropriate genetic testing.

Given the multisystem nature of the disorder, a multidisciplinary management approach is warranted. In our patient, physical therapy was initiated early to support motor development, focusing on core muscle strengthening, prevention of secondary joint injury, and improvement of head control. Orthopedic follow-up is essential given the bilateral hip dislocation and thoracolumbar kyphoscoliosis; conservative management with physiotherapy and orthotic support is typically the first-line approach in infants, with surgical intervention considered if conservative measures prove insufficient. Skin fragility and joint hypermobility require avoidance of high-impact activities and use of joint protective strategies. The observed relative underweight (−2.45 SD) at 10 months may reflect inadequate caloric intake secondary to muscular hypotonia, even though overt feeding difficulties were not reported. Therefore, early nutritional assessment and monitoring of feeding efficiency are recommended as part of the comprehensive care for infants with severe hypotonia associated with collagenopathies. Periodic cardiac surveillance with echocardiography is recommended given the identified mitral valve prolapse, with increased frequency of follow-up if hemodynamic significance increases. Respiratory monitoring is advised in light of the early pneumonia episode, even in the absence of chronic respiratory involvement. Neurodevelopmental assessment and speech therapy are indicated given the observed delays in motor and vocal milestones.

Type I collagen is the most abundant structural protein in humans, accounting for approximately 30% of total body protein and 80% of fibrillar collagen in the extracellular matrix. It is a heterotrimer composed of two α1(I) chains, encoded by *COL1A1*, and one α2(I) chain, encoded by *COL1A2*. These polypeptide chains assemble into a right-handed triple helix characterized by a repeating Gly–X–Y motif, in which glycine occupies every third position, while proline and 4-hydroxyproline are commonly found in the X and Y positions. This structure, stabilized by hydrogen bonding and post-translational modifications such as hydroxylation and glycosylation, confers high thermal stability and mechanical strength to collagen fibrils [13].

The molecular mechanism of the missense variant c.2050G>A (p.Glu684Lys) has not been definitively established. The substitution of the negatively charged Glu684 residue with positively charged lysine is predicted to disrupt the local electrostatic balance within the helical domain, potentially suppressing intrachain or interchain salt bridge formation and causing partial helix destabilization. Charged amino acids play a critical role in triple helix assembly and fibrillogenesis, and similar charge-disrupting effects, including delayed procollagen secretion and structurally unstable extracellular matrix fibrils, have been described for other missense variants in the collagen helical domain [14,15]. These statements are hypothetical; further functional studies are needed for a complete understanding of the interaction mechanisms.

Collagens carry out critical extracellular matrix functions by interacting with cell receptors, including integrin α2β1, which recognizes fibrillar collagens via the strictly conserved GFOGER motif. The glutamate residue within this motif directly chelates the metal ion in the MIDAS domain of integrin α2β1, and even a conservative substitution of glutamate to aspartate has been shown to abolish binding due to insufficient side-chain length [16,17]. Notably, Glu684 in the current MANE/HGVS numbering of the COL1A1 pro-α1 chain corresponds to the glutamate residue at position E within the well-characterized GFOGER integrin-binding motif (G680–F681–O682–G683–E684–R685), which corresponds to position 506 in the classic mature/triple-helical numbering used in the integrin–collagen structural literature. Although the p.Glu684Lys substitution is predicted to disrupt integrin–collagen interaction, in vivo impairment of this interaction has not been experimentally confirmed for this particular variant. Consequently, the functional consequences remain hypothetical.

Taken together, these findings demonstrate that pathogenic variants in the *COL1A1* gene give rise to a broad and continuous spectrum of overlapping phenotypes encompassing neurological, orthopedic, cutaneous, cardiovascular, and respiratory manifestations. The recurrent homozygous *COL1A1* variant c.2050G>A, p.(Glu684Lys) has been reported predominantly in families of Saudi Arabian origin, although it was also recently identified in an Australian neuromuscular disease cohort (Marchant et al., 2024) [7,8,9]. The present patient, however, is of Avar ethnicity, representing a geographically and ethnically distinct population. Haplotype analysis was not performed in the current study; therefore, it remains unknown whether this variant represents a shared founder haplotype or arose as an independent mutational event. This constitutes a limitation of the present study and warrants further investigation, including haplotype analysis in future cases. The c.2050G>A (p.(Glu684Lys)) variant identified in our patient is associated with an arthrochalasia-like EDS phenotype that overlaps with previously reported cases and further refines the phenotypic characterization of *COL1A1*-related disorders. These data emphasize the complexity of genotype–phenotype correlations in type I collagenopathies and underscore the need for continued accumulation of well-characterized clinical and molecular data to refine disease classification and improve diagnostic and management strategies.

## 5. Conclusions

This study describes one of the few reported cases worldwide of autosomal recessive arthrochalasia-like Ehlers–Danlos syndrome phenotype caused by a homozygous *COL1A1* variant (p.(Glu684Lys)). The clinical presentation observed in our patient supports substantial phenotypic variability associated with this variant and further refines its phenotypic characterization. Our findings indicate that the phenotype aligns more closely with arthrochalasia-like EDS presentation than with classical EDS, despite the unusual autosomal recessive inheritance pattern. This case also highlights the importance of considering collagenopathies in the differential diagnosis of infants presenting with congenital hypotonia, particularly when neuromuscular disorders are initially suspected. Further accumulation of clinical and molecular data is required to better define the phenotypic spectrum and improve diagnostic accuracy for COL1A1-related disorders.

## Figures and Tables

**Table 1:** Clinical characteristics of the current patient and previously reported cases with Ehlers–Danlos syndrome associated with a homozygous missense variant c.2050G>A (p.(Glu684Lys)) in the *COL1A1* gene (NM_000088.4). “+”—feature present; “−”—feature absent.

Clinical Characteristics	Current Case	Alazami AM et al. (2016) [7]		Almatrafi A et al. (2020) [8]		Marchant et al. (2024) [9]
		Family 13	Family 14	Family 1		Family 1
Age at last follow-up	10 months	6 months	7 months	Not reported	Not reported	Not reported
Skin involvement (including skin hyperextensibility)	+	+	+	+	+	Not reported
Easy skin bruising	-	-	+	+	+	Not reported
Wrinkled skin	+	+	+	-	+	Not reported
Joint hypermobility	+ (Beighton 8/9)	+	+	+ (Beighton 5/9)	+ (Beighton 5/9)	Not reported
Congenital hip dislocation/subluxation	+	-	+	-	-	Not reported
Fractures	-	+ Recurrent fracture	+ Recurrent fracture (generalized osteopenia)	-	-	Not reported
Congenital hypotonia	+	+	+	-	-	+ Presumed (Neuromuscular disorders cohort)
Motor milestones	Delayed	Delayed	Delayed	Not reported	Not reported	Not reported
Respiratory failure	-	+ at birth	+ at birth	-	-	Not reported
Respiratory support required	-	Not reported	Not reported	-	-	Not reported
Pneumonia history	+ (at 3 months)	Not reported	Not reported	-	-	Not reported
Decreased deep tendon reflexes	+	+	+	-	-	Not reported
Kyphosis/scoliosis	+	-	+	-	-	Not reported
Cardiovascular involvement	Mitral valve prolapse with hemodynamically insignificant mitral regurgitation (grade 1+)	-	-	-	Not reported	Not reported
Craniofacial dysmorphism	+	+	+	-	-	Not reported
Speech/vocal development	The first syllables appeared at 9 months	Not reported	Not reported	-	-	Not reported

## Data Availability

The original contributions presented in this study are included in the article. Further inquiries can be directed to the corresponding author.
